# Viscoelastic Hemostatic Assays in the Management of Trauma-Induced Coagulopathy: A Clinical Update

**DOI:** 10.3390/jcm15010012

**Published:** 2025-12-19

**Authors:** Daniele Natalini, Rikardo Xhemalaj, Simone Carelli

**Affiliations:** 1 Dipartimento di Scienze dell’Emergenza, Anestesiologiche e della Rianimazione, Fondazione Policlinico Universitario A. Gemelli IRCCS, 00168 Rome, Italy; daniele.natalini@policlinicogemelli.it (D.N.); rikardoxhemalaj@gmail.com (R.X.); 2 Dipartimento di Scienze Biotecnologiche di Base, Cliniche Intensivologiche e Perioperatorie, Università Cattolica del Sacro Cuore, 00168 Rome, Italy

**Keywords:** trauma-induced coagulopathy, viscoelastic hemostatic assays, damage control resuscitation, hemostatic resuscitation

## Abstract

The recognition of trauma-induced coagulopathy (TIC) as an endogenous response to traumatic injuries rather than a consequence of therapeutic interventions has shifted the clinical approach toward an early and physiologically based hemostatic resuscitation. Prompt identification and correction of fibrinolysis and fibrinogen level derangements, dysregulated thrombin generation, and platelet dysfunction represent the cornerstones of the treatment strategies. Currently available viscoelastic hemostatic assays (VHAs) are point-of-care devices able to rapidly assess the phases of clot initiation, propagation, stabilization, and degradation, as well as isolate the contribution of specific elements—e.g., fibrinogen—to the coagulation process in fully automated analyses by multi-channel single-use cartridges. As a result, in the last decade, VHAs have been widely investigated as tools to implement individualized protocols of hemostatic resuscitation. Current guidelines support their use to optimize transfusion load in a goal-directed strategy. Nevertheless, contrasting evidence has emerged regarding the improvement in main clinical outcomes induced by the VHA-based algorithm of hemostatic resuscitation compared with those guided by conventional coagulation tests, and their place in the management of this peculiar population is still a matter of debate. We propose a narrative review ranging from TIC physiopathology as a proper substrate for viscoelastic diagnostic technique, through the strengths and weaknesses of VHAs, to their application in clinical practice.

## 1. Introduction

Coagulopathy is a pillar of life-threatening post-traumatic bleeding, combined with metabolic acidosis, hypothermia, and hypocalcemia in the “diamond of death”. Uncontrolled hemorrhage may account for up to 25% of injury-related deaths, many of them having a coagulopathic component; similarly, more than 30% of severe trauma patients admitted to the emergency department are in a coagulopathic state [1]. Trauma-induced coagulopathy (TIC) refers to the evolving patterns of hemostatic derangements attributable to trauma; while the immediate post-traumatic hours are characterized by a profile of hypocoagulability resulting in a bleeding phenotype (early TIC), a dynamic and progressive transition to a subsequent phase of hypercoagulable state occurs, with an increased risk of thrombotic events (late TIC). Prompt characterization of the underlying hemostatic disorders represents a cornerstone in the treatment of coagulopathic trauma patients, augmenting damage control resuscitation [2].

Viscoelastic hemostatic assays (VHAs) are point-of-care tools that may provide, in a few minutes, quantitative and qualitative assessments of the phases of clot initiation, propagation, stabilization, and degradation, namely the speed of clot formation, its maximum strength/firmness, and the magnitude of fibrinolysis, as well as additional measurements, including the specific contribution of fibrinogen [3]. The introduction and spread of VHAs have affected the clinical practice of hemostatic resuscitation. Overcoming some of the limitations of conventional coagulation tests (e.g., the time to collect results from multiple tests and the inability to study fibrinolysis) and identifying the need for specific blood component supplementation, they enable fast decision-making and affect blood product transfusion practices, including massive transfusion protocols [4]. Indeed, in the past decade, the adoption of VHA-based rather than conventional coagulation test (CCT)-based individualized protocols of hemostatic resuscitation has been associated with a reduction in platelet and plasma consumption, even though contrasting evidence has emerged regarding the impact on main clinical outcomes [5]. Current guidelines [6] recommend the implementation of goal-directed strategies, including viscoelastic testing, to guide the hemostatic resuscitation of trauma patients.

In this narrative review, we aim to provide a clinically oriented update in light of the latest evidence and to explore the strengths and limitations of VHAs in trauma care. From physiopathological background to current indications, we summarize the place for VHAs in the assessment of trauma-induced coagulative derangements and guidance of hemostatic resuscitation.

## 2. Methods

A comprehensive literature search was performed across the PubMed, Embase, and Web of Science databases. The search employed a combination of Medical Subject Headings (MeSH) and free-text terms, including “viscoelastic hemostatic assays”, “thromboelastography”, “rotational thromboelastometry”, “trauma-induced coagulopathy”, “hemostatic resuscitation”, “damage control resuscitation”, “major trauma hemorrhage”, and “massive transfusion protocol”. The search was restricted to articles written in English and published between 2000 and 2025. Eligible articles included randomized controlled trials, observational cohort studies, systematic reviews, relevant clinical guidelines, and expert commentaries that provided data or expert perspectives on hemostatic resuscitation and the use of VHAs in trauma patients. Reference lists from key articles and relevant guidelines were also reviewed to ensure the inclusion of important studies not captured by the initial database search.

## 3. Viscoelastic Hemostatic Assays

Viscoelastic hemostatic assays provide a dynamic bedside assessment of global hemostasis using whole blood under low-shear conditions. Unlike CCTs, which isolate single components of the coagulation cascade and often suffer from delayed turnaround, VHAs capture in real time the entire process of clot initiation, propagation, stabilization, and breakdown. The first prototype was described by Hartert in 1948, and technological refinements over subsequent decades have established VHAs as valuable tools in perioperative and critical care medicine [7,8].

**Figure 1:**
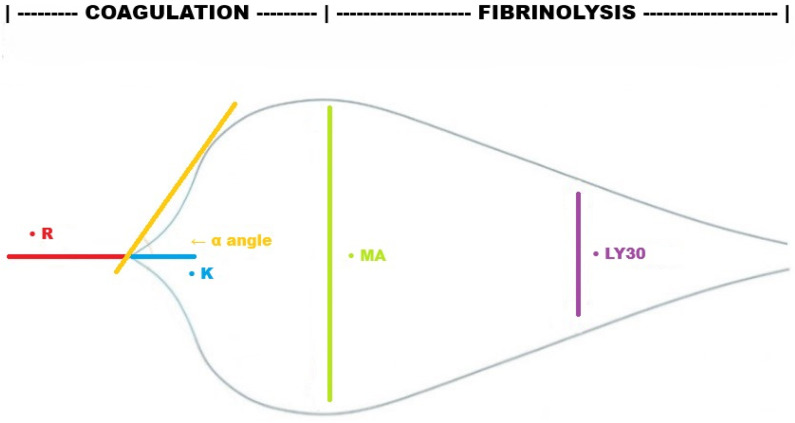
Standard TEG tracing illustrating clot initiation (R), propagation (K and α-angle), stabilization (MA) and degradation (LY30). R, reaction time; K, kinetics; MA, maximum amplitude; LY30, lysis index at 30 min.

Even though several devices have been developed in recent years, two traditional platforms dominate current clinical practice: for thromboelastography, TEG (Haemonetics Corporation, Braintree, MA, USA) and for rotational thromboelastometry, ROTEM (Tem Innovations GmbH, Munich, Germany). Both provide tracings that illustrate the kinetics of clot formation, though their operating principles differ slightly. In the TEG system, a pin on a torsion wire is suspended in a cylindrical cup containing a whole-blood sample, which oscillates under constant force. As the viscoelastic strength of the clot increases, progressively more rotation is transmitted to the torsion wire, and this is detected by an electromagnetic transducer. In the latest version of the device, using LED illumination, an infrared detector measures vertical motion of the coagulating blood meniscus, and the changes in resonance when the blood coagulates are recognized by the analyzer and converted to a graphical image (Figure 1). Conversely, in ROTEM devices, a cup of whole blood remains fixed in a heating block while a pin suspended on a ball bearing mechanism oscillates. As the viscoelastic strength of the clot increases, the rotation of the pin is progressively impeded, and this is detected optically by a charge-coupled device image sensor system. The latest-generation models of these point-of-care coagulation monitoring systems (TEG 6s and ROTEM Sigma, respectively) perform fully automated viscoelastic analyses by multi-channel single-use cartridges [3,9]. Both TEG and ROTEM platforms provide different key hemostatic parameters, with specific clinical relevance (Table 1), and offer a range of assay types, each using proper reagents to initiate clotting and isolate individual hemostatic components (Table 2 and Table 3). In each assay, the devices assess clot initiation, propagation, stabilization, and degradation through the following parameters for TEG/ROTEM: reaction time (R)/clotting time (CT), kinetics (K)/clot formation time (CFT) and α-angle, maximum amplitude (MA)/maximum clot firmness (MCF), lysis at 30 min (LY30), and maximum lysis (ML) (Figure 1). The adjunct of specific activators/inhibitors enables the assessment of specific contributors to the hemostatic pathway, which represent therapeutic targets, as fibrinogen in citrated functional fibrinogen (CFF)/FIBTEM. Their ease of use has accelerated the integration of VHAs across several clinical domains. Trauma and major hemorrhage management remain the best-studied applications [10], but evidence also supports their role in cardiac surgery [11], obstetric hemorrhage [12], liver surgery [13], and sepsis [14,15]. Importantly, VHA-guided transfusion strategies consistently reduce exposure to allogeneic blood products, although demonstration of a survival advantage is still lacking [9]. To note, we chose to focus on the TEG and ROTEM devices only, and we herein refer to TEG 6s and ROTEM Sigma versions even though not specified.

## 4. Trauma-Induced Coagulopathy

### 4.1. Epidemiology and Clinical Landscape

Trauma-induced coagulopathy is a frequent and early derangement of hemostasis, affecting 25–35% of severely injured patients upon hospital admission; uncontrolled bleeding has been reported to cause 25% of all injury-related deaths and 40–80% of potentially preventable deaths. Its presence independently predicts the need for transfusion, multi-organ dysfunction, and early death, even after adjustment for confounding factors such as dilution, hypothermia, and acidosis [17,18]. Clinical drivers include severe tissue disruption (including penetrating mechanisms) and hypoperfusion, shock, metabolic acidosis, extended prehospital times, and liberal crystalloid resuscitation; hypocalcemia, frequently induced by shock and citrate-based transfusions, has been added to the classical “lethal triad”, redefining it as the “lethal diamond” [19].

Trauma-induced coagulopathy is sustained by early dysregulated thrombin generation, hyperfibrinolytic state, and platelet dysfunction, posing a risk of progression to disseminated intravascular coagulation; however, TIC evolves dynamically, and while the early phase is dominated by hypocoagulability with uncontrolled bleeding (early TIC), the later stages may manifest hypercoagulability and thromboembolic risk (late TIC) [2,20].

### 4.2. Physiopathology: From Tissue Injury to Hemostatic Derangements

The physiopathology of TIC involves a dynamic interplay between endothelial dysfunction, dysregulation of coagulation pathways, tissue hypoperfusion, and systemic inflammation. Endothelial disruption is an early and pivotal event: tissue injury and shock synergistically activate endothelial cells, exposing tissue factor and initiating the coagulation pathway, a process that becomes dysregulated under hypoxic and inflammatory conditions. Systemic inflammation triggered by damage-associated molecular patterns liberated from injured cells amplifies the cytokine network and activates neutrophils, which release extracellular traps, exacerbating endothelial injury and coagulation disturbances [21]. As a result, bleeding with ongoing thrombin generation induces consumption coagulopathy. Traumatic injuries disrupt the endothelial glycocalyx, also resulting in the release of endogenous heparin-like substances—syndecan-1 and heparan sulphate—which increases vascular permeability and promotes endogenous anticoagulant pathways [22,23]. A critical downstream alteration involving the physiological anticoagulant protein C pathway is induced. Tissue injury and hypoperfusion accelerate thrombin binding to thrombomodulin, generating activated protein C (aPC), which inactivates FVa and FVIIIa as well as suppresses plasminogen activator inhibitor-1 (PAI-1); this cascade impairs thrombin activity, destabilizes fibrin, and abrogates protective endothelial signalling [24]. Hyperfibrinolysis is a prominent feature of TIC, mainly driven by an excessive release of tissue plasminogen activator (tPA) from the injured endothelial cells and insufficient inhibition by PAI-1, resulting in premature clot breakdown. Conversely, an early inadequate fibrinolytic activity could also occur, resulting in fibrinolysis shutdown with microvascular thrombosis; both phenotypes carry poor prognostic significance. Loss of thrombin-activatable fibrinolysis inhibitors further destabilizes this balance [25,26]. Fibrinogen depletion occurs rapidly due to consumption and dilution, undermining clot stability. Concurrently, platelet dysfunction is another hallmark of TIC, where trauma, hypoperfusion, and systemic inflammation impair platelet adhesion, activation, and aggregation, despite normal or elevated platelet counts [27]. Even though TIC is an impairment of hemostasis biochemically evident prior to and independent of the development of shock-related acidosis, hypothermia, and hemodilution, these factors worsen its profile and precipitate its effects. For instance, iatrogenic factors can exacerbate dilutional coagulopathy, such as fluid resuscitation and transfusion of packed red blood cells (pRBCs) without adequate hemostatic components. The overall result is a systemic state where clot formation is impaired, clots that do form are unstable and prone to lysis, and bleeding becomes difficult to control.

The close network between endothelium, hemostasis, and inflammation pathways is responsible for the dynamic transition to the subsequent phase of hypercoagulability of TIC. Following extensive tissue and endothelial disruption, tissue factor is exposed and complexed with FVII, promoting thrombin generation. The release of damage-associated molecular patterns and other proinflammatory cytokines activates neutrophils and macrophages, which amplify clot formation and endotheliopathy, and platelet adhesion on the injured endothelium and aggregation contribute to the propagation of the self-potentiating hemostatic and inflammatory process. In addition, hyperfibrinolysis is reversed by a PAI-1 rise, usually occurring 2 to 10 h after traumatic injury. Hence, late TIC (usually > 24 h after injury) is dominated by a hypercoagulable state, which may result in macro- and microvascular thrombosis, leading to thromboembolic events and multiple organ failure [2]. Interestingly, early and late TIC are not mutually exclusive, and the transition from hypocoagulability to hypercoagulability may occur within minutes or hours or be delayed for days. In such a setting, VHAs may be crucial due to their capability of assessing the entire hemostatic process, on the one hand, and isolating its components, on the other.

### 4.3. Diagnosis

The diagnosis of TIC may be challenging, requiring the integration of clinical and laboratory data; given the vast array of coagulation changes in patients with traumatic injury, defining TIC with a single laboratory measurement may be misleading. Moreover, the most relevant clinical goal should be the prediction of TIC in the prehospital setting or as soon as possible at hospital admission in order to implement patient-targeted resuscitation. Hence, several scores merging clinical and laboratory data have been developed to stratify risk and guide management. The Coagulopathy of Severe Trauma score, derived from clinical and laboratory variables, predicts the likelihood of acute traumatic coagulopathy at admission [28]. The Trauma-Induced Coagulopathy Clinical Score incorporates mechanism of injury, hemodynamic status, and laboratory data, demonstrating utility in early prehospital prediction of TIC [29].

Trauma-induced coagulopathy is usually defined by an international normalized ratio (INR) > 1.2, with an INR > 1.5 suggesting severe coagulopathy. Apart from INR, thresholds for other CCTs are less well-established. An activated partial thromboplastin time (aPTT) > 35 s, a Clauss fibrinogen < 1.3 g/L, and markers of fibrinolysis, such as elevated D-dimers, may provide baseline data, but contrasting evidence has emerged regarding their correlation with bleeding risk and clinical outcomes [30]. CCTs were originally developed to standardize monitoring of oral anticoagulant therapy across laboratories rather than to screen for coagulation disorders, hence having many limitations affecting clinical practice in this particular setting. Of note, they reflect only the contribution of plasma proteins to clot formation, without regard for the central role of platelets (erythrocytes and platelets are eliminated during the centrifugation process). They can identify only the early phase of the coagulation process and cannot recognize the activation of endogenous anticoagulatory pathways [31,32]. Although there is no commonly accepted viscoelastic definition of TIC, VHAs overcome these limitations by delivering real-time information on clot initiation, propagation, strength, and lysis. Once trauma-induced coagulopathy is identified, the challenge shifts from diagnosis to dynamic monitoring. In this setting, viscoelastic hemostatic assays detect coagulopathy phenotypes invisible to standard tests and enable real-time, individualized correction of coagulation derangements according to transfusion needs.

## 5. Clinical Phenotypes

**Figure 2:**
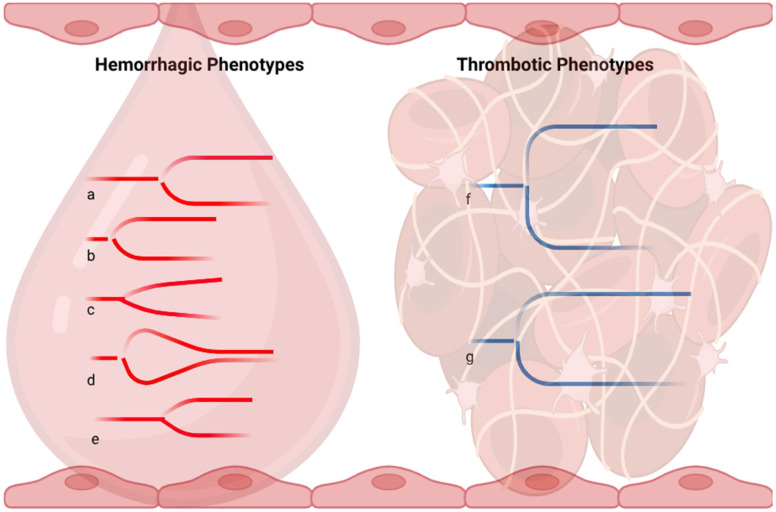
Infographic representation of the main viscoelastic phenotypes of trauma-induced coagulopathy. a, clotting factor deficiency (anticoagulant therapy); b, decreased platelet function and/or decreased fibrinogen level; c, decreased fibrinogen level at a specific test; d, hyperfibrinolysis; e, clotting factors deficiency and decreased platelet function/decreased fibrinogen level; f, increased platelet function and/or increased fibrinogen level; g, fibrinolysis shutdown.

The main TIC alterations and profiles detectable by VHAs include hypocoagulability, hypercoagulability, fibrinolysis derangements, hypofibrinogenemia, and platelet dysfunction (Figure 2).

### 5.1. Hypocoagulability and Hypercoagulability

Hypocoagulability is an early signature of TIC, reflecting impaired thrombin generation secondary to tissue hypoperfusion, consumption of clotting factors, and dilution by resuscitation fluids [33,34]. Viscoelastic hemostatic assays consistently demonstrate prolonged clot initiation times (R/CT), depressed clot propagation (prolonged K/CFT and low α-angle), and overall reduced clot strength (low MA/MCF). The ability of VHAs to diagnose a specific deficit in specific coagulation factors is, however, relatively poor and can also reflect hypofibrinogenemia and/or hyperfibrinolysis [35]. On the other hand, hypercoagulability is a subacute finding—although it has been described as early as minutes to hours after trauma—correlated with accelerated thrombin generation predisposing to venous thromboembolism [36]. Currently, little is known about the timing of this transition, as physiopathology is likely multifactorial, involving systemic inflammation triggers [2].

### 5.2. Hypofibrinogenemia

Fibrinogen is the first coagulation factor to reach critical thresholds after major trauma [37], and the accuracy of VHAs is excellent for both diagnosing fibrinogen deficit and monitoring the increase in fibrinogen levels after supplementation. Moreover, VHAs can accurately isolate the contribution of fibrinogen and platelets to clot firmness [38,39].

### 5.3. Fibrinolysis Derangements

Among TIC phenotypes, hyperfibrinolysis carries the highest risk of early death. Hyperfibrinolysis at hospital admission is an independent predictor of mortality within six hours of injury, with lethality rates exceeding 70% despite aggressive resuscitation, even though the real proportion of patients experiencing trauma-associated hyperfibrinolysis is unknown [40,41]. Conventional coagulation tests are blind to fibrinolysis; only VHAs allow direct recognition and stratification of fibrinolytic activity at the bedside, informing timely administration of tranexamic acid. Beyond hyperfibrinolysis, VHAs have uncovered an equally ominous phenotype: fibrinolysis shutdown, a well-recognized fibrinolytic pattern in trauma, affecting a number of severely injured patients, even in the early phase [42]. Clinically, the shutdown is associated with organ failure, thromboembolic events, and late mortality. Interestingly, the transition from early to late TIC can invert the fibrinolysis status. The ability of VHAs to discriminate between hyperfibrinolysis and fibrinolysis shutdown is clinically crucial, as both states are pathologic but demand opposite therapeutic approaches.

### 5.4. Platelet Dysfunction

Platelets play a central role in TIC, and VHAs provide global readouts of their contribution to clot firmness. Experimental trauma models have demonstrated impaired platelet aggregation within minutes of shock, regardless of the count, due to receptor shedding and mitochondrial dysfunction [43]. While the detection of platelet contribution to clot firmness can be assessed with VHAs, their function independent of platelet count and correlation with the transfusion trigger remains hard to assess. There are different phenotypes of platelet dysfunction following trauma, and the number of patients with pre-trauma use of antiplatelet agents is increasing [44]. The utility of point-of-care assays, such as TEG Platelet Mapping, in the detection or exclusion of pre-injury antiplatelet agent treatment is limited [45]. As diagnostic cut-offs for pathologic platelet dysfunction after traumatic injury have not been established, distinguishing pharmacologic from trauma-induced platelet receptor hypofunction is not easy.

## 6. Stepwise Interpretation of Viscoelastic Hemostatic Assays for Goal-Directed Therapy

The use of a variety of reagents accounts for the differences in tracing times. Rapid TEG and EXTEM, which are activated by tissue factor, guarantee a shorter turnaround time. Moreover, amplitude variables at 5 min after coagulation time [A5] and 10 min after coagulation time [A10] are provided by EXTEM, while early detection of fibrinogen-mediated clot firmness at 5 min [A5] and 10 min [A10] is assessed by both TEG and ROTEM. These enable rapid monitoring and early proper replacement therapies; hence, they have been widely used in trauma settings and also in the algorithm of goal-directed therapy [35,46].

### 6.1. Prolonged R/CT

TEG reaction time and ROTEM clotting time assess the initiation of the coagulation process, which is influenced by coagulation factors, endogenous anticoagulants, and medications such as heparin, direct oral anticoagulants, or vitamin K antagonists. Taking into account clinical severity and individual variability, triggers for intervention may be considered: for TEG, R > 12 min or 3 min (citrated kaolin and citrated rapid test, respectively) and for ROTEM, CT > 85 s or 205 s (EXTEM and INTEM, respectively). To note, a longer R/CT is not always indicative of altered thrombin generation, as low fibrinogen levels alone may account for this alteration. Gratz and colleagues showed in an experimental study that administering fibrinogen concentrate can considerably shorten R/CT, more than prothrombin complex concentrates (PCCs) [42,43]. Prothrombin complex concentrates and fresh frozen plasma (FFP) are indicated when there is clear evidence of impaired thrombin generation, i.e., when prolonged R/CT is associated with CFF or FIBTEM simultaneously showing a normal fibrin polymerization amplitude [42,44]. In addition, Hofmann and colleagues recently conducted an in vitro experimental study on a small number of healthy volunteers comparing thrombin generation assays with VHA-derived parameters of clot initiation. The authors demonstrated that upregulated thrombin generation parameters after PCCs spiking were not detected by CT, activated clotting time, or standard tests, thereby questioning the concept of VHAs as a guide for PCCs administration [45].

### 6.2. Low MA/MCF with Normal Fibrinogen Contribution

Maximum clot strength results from the formation of the fibrin network, FXIII levels, and platelet contribution [47]. A reduced clot amplitude (CA) in TEG-MA or ROTEM-MCF associated with normal CA in the fibrin polymerization tests (CFF-MA > 20 mm and FIBTEM > 12 mm) may indicate thrombocytopenia, and platelet transfusions could be considered in bleeding patients [35].

### 6.3. Low MA/MCF with Reduced Fibrinogen Contribution

Thromboelastographic CFF and ROTEM-FIBTEM provide an early estimate of fibrinogen concentration and contribution to the hemostatic process [35,48]. The target after fibrinogen replacement should be >20 mm for CFF and >12 mm for FIBTEM. This difference in cut-offs is primarily due to the role of the platelet inhibitor adopted: cytochalasin D, applied in the ROTEM-FIBTEM test, is more efficient compared to the GPIIb/IIIa inhibitor abciximab used in the TEG-CFF test [16,47].

### 6.4. Hyperfibrinolysis and Fibrinolysis Shutdown

Hyperfibrinolysis is defined for TEG as a reduction of >3% of the MA (LY30) and for ROTEM as a maximum lysis of >15% [49]. There is evidence of increased mortality in trauma patients with documented hyperfibrinolysis [41,50]. Another VHAs sign for strong profibrinolytic activation is a complete “flat line” in the CFF/FIBTEM [51]. These circumstances can justify the administration of antifibrinolytic agents such as tranexamic acid. The identification of absent fibrinolytic activity—undetectable LY30 or ML—defines fibrinolysis shutdown.

### 6.5. High Clot Amplitude

A hypercoagulable trend may be observed, especially in late TIC; it is reasonable to withhold coagulation support therapy and/or consider the timing of antithrombotic prophylaxis [47].

### 6.6. Platelet Function Testing

The predictive value of point-of-care platelet function testing is still not well demonstrated. There is no consensus, and current guidelines do not recommend using it as guidance for platelet transfusion strategies [52,53].

## 7. Hemostatic Resuscitation: The Place for Viscoelastic Hemostatic Assays

### 7.1. Current Evidence

In the past decade, a growing number of studies have evaluated whether VHA-based resuscitation protocols could provide clinical advantages over CCT-based strategies in patients with TIC. The hypothesis is that point-of-care, physiology-based hemostatic monitoring enables a faster and more targeted correction of coagulation abnormalities, thereby reducing unnecessary transfusions and improving outcomes.

In 2015, Nardi et al. [5] prospectively observed that the implementation of a ROTEM-based protocol—the Early Coagulation Support—in two Italian trauma centers was associated with a marked and significant reduction in blood product consumption, namely plasma and platelets. In 2016, Gonzalez and colleagues [54] conducted the first pragmatic randomized controlled trial (RCT) to address these issues. One hundred eleven patients with major trauma who met criteria for massive transfusion protocol activation were enrolled, and the TEG-guided protocol resulted in a survival benefit compared with targeted therapy based on CCTs. Overall, patients of the CCT-guided group received more plasma, platelet, and cryoprecipitate transfusions in the early phase of resuscitation; more ICU-free days and ventilator-free days were observed for survivors in the TEG-guided group. The ITACTIC trial [55] enrolled 396 trauma patients already receiving hemostatic resuscitation according to local empiric massive hemorrhage protocols (pRBCs/FFP/platelets in a 1:1:1 ratio) and randomized them in a 1:1 ratio to the VHAs or CCTs groups; the two strategies were test-driven every four units of RBCs transfused, according to the TACTIC algorithms [35]. The primary outcome—patients alive and free from massive transfusion at 24 h—did not differ significantly between groups; a reduction in 28-day mortality in the VHAs arm was observed in patients with severe traumatic brain injury. Interestingly, 67% of patients in the VHAs group received study interventions—1.8 times more than in the CCTs group—and exhibited fewer uncorrected coagulopathies at 3 h, confirming the documented evidence of coagulation deficits not detected by CCTs. The authors recorded a lower-than-expected prevalence of coagulopathy in the study population overall, likely due to early prehospital optimization and transfusions before randomization. Consistently, Abraham et al. [56] provided a commentary on the ITACTIC trial, questioning why no survival benefit was observed with VHA-guided protocols. The authors noted that most enrolled patients were not frankly coagulopathic and had minimal transfusion needs, which likely diluted any potential effect. They also criticized the hybrid design, where empirical transfusion preceded algorithmic correction, possibly masking the benefits of early VHA-driven decisions. Overall, they concluded that future studies should target patients with severe coagulopathy and apply an early, stepwise VHA-based resuscitation algorithm to truly assess the clinical impact. A subgroup analysis, including the ITACTIC population co-enrolled into another prospective observational study and monitored with both VHAs and CCTs, observed that the viscoelastic hemostatic assay group was more likely to receive goal-directed treatment than the standard group; the replacement therapies were delivered earlier when based on VHAs rather than CCTs [57]. In 2023, David and colleagues performed a propensity score-matched analysis of severely injured patients, comparing a ROTEM-guided hemostatic algorithm with CCT-guided care. The VHAs group had a substantially higher proportion of patients alive and free of massive transfusion at 24 h and received far fewer blood products with a reduction in resuscitation costs, but there was no difference in overall 24 h or 28-day mortality [58]. Brunskill et al. [59] included 18 RCTs with a total of 5041 participants in a recent meta-analysis, trying to address the most relevant questions regarding blood transfusion strategies for major bleeding in trauma. Unfortunately, only two RCTs [54,55] were included regarding the goal-directed blood transfusion strategy of VHAs versus CCTs. The authors found little or no difference in all-cause mortality at 24 h between VHAs and CCTs (RR: 0.85, 95% CI: 0.54 to 1.35; 1 study, 396 participants; low-certainty evidence). Moreover, their effects on all-cause mortality at 30 days remain uncertain (RR: 0.75, 95% CI: 0.48 to 1.17; 2 studies, 506 participants; very-low-certainty evidence). No difference was described between VHAs and CCTs in the impact on total thromboembolic events at 30 days (RR: 0.65, 95% CI: 0.35 to 1.18; 1 study, 396 participants; moderate-certainty evidence).

In a recent retrospective analysis on about 1500 patients admitted to seven trauma centers in the United States, Murali et al. [60] developed a “TIC score”, with 1 point assigned for each of three abnormal TEG/ROTEM parameters (R/CT, α-angle, and MA), and observed a strong association between VHAs abnormalities (defined by a TIC score ≥ 1) and both hospital mortality (53% increase per point increase in the score) and pRBC transfusion volume (25% per point increase).

Some concerns have been raised about inter- and intra-hospital variability in VHAs results, even though a strong within-device reproducibility for the TEG 6S machine has been observed in trauma patients [61].

Collectively, the body of available evidence investigating VHAs in this patient population supports a pragmatic benefit in terms of transfusion efficiency and process metrics, rather than definitive improvements in outcomes.

### 7.2. Guidelines and Clinical Practice

In 2023, *Critical Care* published the European guidelines for the management of major bleeding and coagulopathy, produced by a study group of leading experts in anesthesiology, emergency medicine, hematology, and intensive care medicine [6]. The cornerstones of treatment in patients with ongoing/at risk of massive hemorrhage are high priority for damage control surgery/procedures and early damage control resuscitation, including hemostatic resuscitation. Coagulation support should be implemented immediately at hospital admission and empirically based on pRBCs, tranexamic acid, and fibrinogen supplementation. Indeed, the role of VHAs in the early antifibrinolytic therapy is still a matter of debate. The potential occurrence of fibrinolysis shutdown, as well as its therapeutic implications, are well known, but there is contrasting evidence dealing with the effects of tranexamic acid on VHAs tracings [62,63,64]. Nevertheless, given the high prevalence and the impact on clinical outcomes of hyperfibrinolysis, the intravenous administration of 1 g of tranexamic acid to trauma patients who are bleeding or at risk of bleeding within 3 h after injury, followed by an 8 h intravenous infusion of 1 g, is recommended without waiting for the viscoelastic assessment. Fibrinogen should be supplemented with 2–4 g of fibrinogen concentrate/cryoprecipitate or, alternatively, with FFP, aiming at FFP/pRBCs of 1:1 [5,65]. For intractably exsanguinating patients, massive transfusion protocols improve outcomes when approximating a 1:1:1 ratio of RBCs, FFP, and platelets [65]. However, after the prompt empirical administration of tranexamic acid, fibrinogen, and pRBCs, the subsequent hemostatic resuscitation is recommended to be test-guided—VHAs or CCTs—and centers are encouraged to develop their own procedural protocols to be activated as soon as possible upon hospital admission. Fibrinogen concentrate or cryoprecipitate should be given further if major bleeding is accompanied by hypofibrinogenemia or VHAs signs of functional fibrinogen deficit; the use of FFP to correct documented hypofibrinogenemia should be avoided [66]. For the supplementation of coagulation factors and to increase thrombin generation, there is no proven superiority of one treatment—FFP and PCCs—over the other [67]. The trigger for the intervention is represented by CCTs alterations and/or viscoelastic evidence of delayed clot initiation, e.g., INR/aPTT > 1.5 times normal and/or TEG CK-R > 12 min, respectively. The administration of FFP or PPCs should always be preceded by fibrinogen supplementation and/or evidence of normal fibrinogen levels or function—CFF or FIBTEM. The same coagulation test—CCTs or VHAs—should be used for diagnosis and monitoring of treatment, repeated after each cycle of replacement therapy and until bleeding control/hemodynamic stabilization. Indeed, current evidence does not support the superiority of one test over the other. The routine use of platelet function devices for platelet function monitoring and/or antiplatelet therapy detection should be avoided due to inadequate standardization of the devices, unavailability of accepted diagnostic cut-offs and therapeutic targets, and possibly limited value of the results in case of low platelet count. Hypocalcemia, metabolic acidosis, and hypothermia should be prevented and promptly treated.

Even though the prompt availability and fast turnaround times are recognized advantages, the use of VHAs may be limited by some real-world implementation requirements and practical issues. First, clinicians need to receive specific training for the use of the device and, importantly, for the interpretation of the tests. Secondly, both devices and reagents/cartridges have cost issues, as these assays are much more expensive than conventional coagulation tests; as a result, for instance, their availability in low-resource trauma centers may be limited. Finally, they need to be properly integrated into the local workflow in an emergency setting.

**Figure 3:**
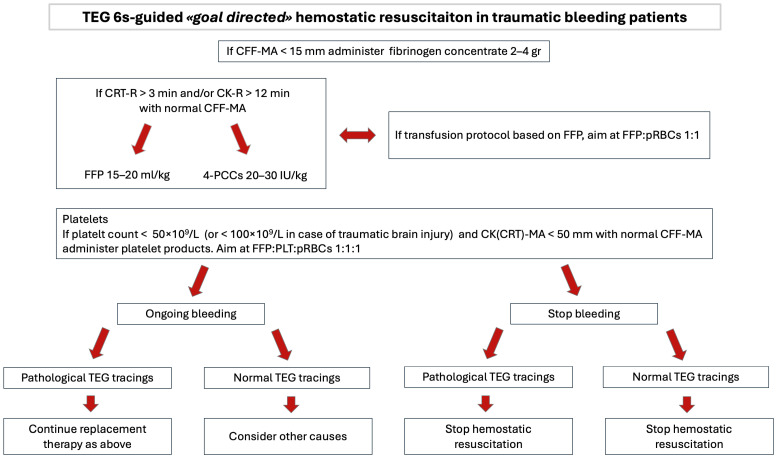
Local TEG 6s-based goal-directed therapy of patients activating massive transfusion protocol after initial empirical hemostatic resuscitation (based on pRBCs, tranexamic acid, and fibrinogen supplementation). TEG, thromboelastography; CFF-MA, citrated functional fibrinogen—maximum amplitude; CRT-R, citrated rapid TEGreaction time; CK-R, citrated kaoline—reaction time; FFP, fresh frozen plasma; 4-PCCs, 4-factors—prothrombin complex concentrates; IU, international units; pRBCs, packed red blood cells; CK-MA, citrated kaoline—maximum amplitude; CRT-MA, citrated rapid TEG—maximum amplitude; PLT, platelets.

Figure 3 illustrates a TEG 6s-based protocol of hemostatic resuscitation after the initial empirical treatment (based on pRBCs, tranexamic acid, and fibrinogen supplementation). This protocol is a locally produced and validated algorithm based on current guideline recommendations and integrated into clinical practice; hence, it has intrinsically limited reproducibility.

### 7.3. Future Perspectives

Based on the available evidence and compared with conventional CCTs, VHAs provide a whole-blood, dynamic, functional assessment of hemostasis and can thus enable goal-directed, individualized therapy rather than “blind” transfusion. However, further research should be conducted to fill the current knowledge gap. While a reduction in blood product use has been reported, major clinical and long-term outcomes (organ failure, functional recovery, thrombosis, and re-bleeding) are less well characterized in VHA-guided protocols. Much of the evidence comes from observational or retrospective studies on a limited patient population, and many published treatment algorithms are center-specific and suffer from the clinical heterogeneity of trauma patients (different mechanisms of injury, severity of shock, timing of damage control surgery, institution-specific massive transfusion protocol activation criteria, and comorbidities), hence limiting their generalizability and reproducibility. The use of VHAs in trauma patients beyond hemostatic resuscitation should also be investigated and defined, including in the perioperative setting, in the subacute and hypercoagulable phases, for the timing of pharmacological prophylaxis of venous thromboembolism, and for the implementation of safe anticoagulation protocols.

## 8. Conclusions

Viscoelastic hemostatic assays offer a direct insight into the hemostatic process, detecting coagulopathy phenotypes invisible to standard tests and hence enabling real-time correction of the underlying derangements, a crucial step toward precision resuscitation. The adoption of VHA-based goal-directed protocols of hemostatic resuscitation has been associated with a reduction in platelets and plasma consumption; however, the contrasting evidence regarding their impact on main clinical outcomes and the well-known real-world limitations of their use (e.g., training and costs) prevent current guidelines from proposing them as first-line diagnostic tools. The integration of VHAs into damage control resuscitation protocols achieves the clinical priority of customizing hemostatic resuscitation, allowing a patient-centered and physiologically guided therapy rather than a blind replacement strategy, thereby supporting the evolution in trauma care.

## Figures and Tables

**Table 1:** TEG and ROTEM parameters.

Phase	TEG	ROTEM	Definition	Clinical Relevance
Clot initiation	R (reaction time) ACT (activated clotting time)	CT (clotting time)	Time to initial clot formation (2 mm amplitude) Time to the beginning of clot formation	Prolonged in coagulation factor deficiency and/or consumption, anticoagulant therapy
Clot propagation	K (kinetics)	CFT (clot formation time)	Time from initial clot to defined firmness (2–20 mm amplitude)	Prolonged in low fibrinogen levels or impaired platelet function
	α-Angle	α-Angle	Angle formed by the tangent line when the clot amplitude reaches 2 mm	Reflects the speed of fibrin build-up; reduced in low fibrinogen levels or, to a lesser extent, in impaired platelet function
Clot stabilization	MA (maximum amplitude)	MCF (maximum clot firmness)	The maximum width of the tracing, representing the maximal clot strength	Decreased in platelet dysfunction or low fibrinogen levels
Clot degradation	LY30 (lysis 30 min after MA, as % of MA)	LI30 (lysis index 30 min after MCF, as % of MCF), ML (maximum lysis, as % of MCF)	Degree of clot breakdown over time	Increased in hyperfibrinolysis Reduced in fibrinolysis shutdown

TEG, tromboelastography; ROTEM, rotational thromboelastometry [3,16].

**Table 2:** TEG assays.

Assay	Activator	Pathway	Main Measurements	Clinical Relevance
Kaolin TEG (CK)	Kaolin	Intrinsic	R, K, α-angle, MA, LY30	Standard TEG test. Assesses clotting factors function, platelet–fibrin interaction, and lysis
Rapid TEG (CRT)	Kaolin + Tissue Factor	Accelerated intrinsic + extrinsic	ACT, R, K, α-angle, MA, LY30	Faster clot initiation for rapid decision-making
Heparinase TEG (CKH)	Kaolin + Heparinase	Intrinsic heparin-neutralized	R, K, α-angle, MA, LY30	Compared to the kaolin test, it differentiates the heparin effect from actual coagulopathy
Functional Fibrinogen (CFF)	Tissue factor + GPIIb/IIIa inhibitor (e.g., abciximab)	Fibrinogen contribution	MA (fibrinogen-only)	Platelets inhibited; assesses the contribution of fibrinogen to clot strength

TEG, thromboelastography; CK, citrated kaolin; R, reaction time; K, kinetics; MA, maximum amplitude; LY30, lysis at 30 min; CRT, citrated rapid TEG; ACT, activated clotting time; CKH, citrated kaolin with heparinase; CFF, citrated functional fibrinogen [3,16].

**Table 3:** ROTEM assays.

Assay	Activator	Pathway	Main Measurements	Clinical Relevance
EXTEM	Tissue Factor	Extrinsic	CT, CFT, α-angle, MCF, ML	Standard ROTEM test. Assesses clotting factors function, platelet–fibrin interaction, and lysis
INTEM	Ellagic Acid	Intrinsic	CT, CFT, α-angle, MCF, ML	Focus on intrinsic pathway defects
HEPTEM	Ellagic Acid + Heparinase	Intrinsic (heparin-neutralized)	CT, CFT, α-angle, MCF, ML	Compared to EXTEM, it differentiates the heparin effect from actual coagulopathy
FIBTEM	Tissue Factor + Cytochalasin D	Fibrinogen contribution	MCF	Platelets inhibited; assesses the contribution of fibrinogen to clot strength

ROTEM, rotational thromboelastometry; CT, clotting time; CFT, clot formation time; MCF, maximum clot firmness; ML, maximum lysis [3,16].

## Data Availability

Not applicable.

## Abbreviations

TIC, trauma-induced coagulopathy; VHAs, viscoelastic hemostatic assays; CCTs, conventional coagulation tests; TEG, thromboelastography; ROTEM, rotational thromboelastometry; R, reaction time; CT, clotting time; K, kinetics; CFT, clot formation time; MA, maximum amplitude; MCF, maximum clot firmness; LY30, lysis at 30 min; ML, maximum lysis; CFF, citrated functional fibrinogen; aPC, activated protein C; PAI-1, plasminogen activator inhibitor-1; tPA, tissue plasminogen activator; pRBCs, packed red blood cells; INR, international normalized ratio; aPTT, activated partial thromboplastin time; PCCs, prothrombin complex concentrates; FFP, fresh frozen plasma; CA, clot amplitude; RCT, randomized controlled trial.
